# Identifying volatile in vitro biomarkers for oral bacteria with proton-transfer-reaction mass spectrometry and gas chromatography–mass spectrometry

**DOI:** 10.1038/s41598-021-96287-7

**Published:** 2021-08-19

**Authors:** Kajsa Roslund, Markku Lehto, Pirkko Pussinen, Kari Hartonen, Per-Henrik Groop, Lauri Halonen, Markus Metsälä

**Affiliations:** 1 Department of Chemistry, University of Helsinki, Helsinki, Finland; 2 Folkhälsan Institute of Genetics, Folkhälsan Research Center, Biomedicum Helsinki, Helsinki, Finland; 3 Abdominal Center Nephrology, University of Helsinki and Helsinki University Hospital, Helsinki, Finland; 4 Clinical and Molecular Metabolism, Faculty of Medicine Research Programs, University of Helsinki, Helsinki, Finland; 5 Oral and Maxillofacial Diseases, University of Helsinki and Helsinki University Hospital, Helsinki, Finland; 6 Department of Diabetes, Central Clinical School, Monash University, Melbourne, VIC Australia

**Keywords:** Mass spectrometry, Oral microbiology, Pathogens, Applied microbiology, Biomarkers

## Abstract

We have measured the volatile fingerprints of four pathogenic oral bacteria connected to periodontal disease and dental abscess: *Porphyromonas gingivalis* (three separate strains), *Prevotella intermedia*, *Prevotella nigrescens* and *Tannerella forsythia*. Volatile fingerprints were measured in vitro from the headspace gas of the bacteria cultured on agar. Concrete identification of new and previously reported bacterial volatiles were performed by a combination of solid phase microextraction (SPME) and offline gas chromatography–mass spectrometry (GC–MS). We also studied the effect of the reduced electric field strength (*E/N*) on the fragmentation patterns of bacterial volatiles in online proton-transfer-reaction time-of-flight mass spectrometry (PTR-ToF-MS). We aimed to discover possible new biomarkers for the studied oral bacteria, as well as to validate the combination of GC–MS and PTR-MS for volatile analysis. Some of the most promising compounds produced include: 1-Methyl-1,2,3,4-tetrahydroisoquinoline (1MeTIQ), indole, and a cascade of sulphur compounds, such as methanethiol, dimethyl disulphide (DMDS) and dimethyl trisulphide (DMTS). We also found that several compounds, especially alcohols, aldehydes and esters, fragment significantly with the PTR-MS method, when high *E/N* values are used. We conclude that the studied oral bacteria can be separated by their volatile fingerprints in vitro, which could have importance in clinical and laboratory environments. In addition, using softer ionization conditions can improve the performance of the PTR-MS method in the volatile analysis of certain compounds.

## Introduction

The oral cavity is home for a wide variety of bacteria, some of which are associated with diseases. Poor oral hygiene can lead to dysbiosis depicted by the accumulation of pathogenic bacteria in the oral cavity. Periodontal diseases are inflammatory diseases in the tooth-supporting tissues causing problems, such as bleeding gums, and eventually, tooth loss. It can also increase the risk for other medical conditions, such as cardiovascular disease and diabetes mellitus^1^. Bacterial infections in the oral cavity can also lead to sepsis, if pathogenic bacteria enter the blood stream in large amounts, for example through a dental abscess. Untreated, oral infections can spread to other parts of the head and neck. Many of the same bacteria are connected to both periodontal disease and dental abscesses. They include species from the genus *Porphyromonas, Prevotella, Tannerella, Treponema, Fusobacterium, Campylobacter* and *Streptococcus*^2,3^.

Oral infections can usually be prevented and treated with good oral hygiene and regular visits to the dentist. In the case of sepsis, however, antibiotics are the only effective treatment. In fact, sepsis is often lethal without them. However, the use of broad-spectrum antibiotics has become problematic, because of the increasing number of antibiotic resistant bacteria^4^. Consequently, in order to decrease the need for antibiotic treatment, it is vital to decrease the prevalence of sepsis. To prevent sepsis induced by oral bacteria, it is important to detect and treat the infections early enough. However, as treatment is often expensive and might not be included, for example, in the universal health coverage, many choose not to treat oral health problems before their effects are already severe. A quick, easy and cost-effective method for detecting oral infections could lower the threshold for patients to seek treatment. Measurement of exhaled volatile compounds produced by pathogenic oral bacteria would be a useful, non-invasive method for such diagnostics.

Different bacterial species produce a vast variety of volatile compounds, some of which are common to many species and some of which are unique to only a few^5^. Identifying bacteria by the volatile organic compounds (VOCs) they produce—their so-called volatile fingerprints—can be quick and easy. Standard culturing to identify bacteria can take days, which is not ideal in the case of severe medical conditions, such as sepsis. Analysis of bacterial VOCs, in contrast, can be performed in a time scale of seconds or minutes, depending on the analysis method. In the case of the oral bacteria, volatile metabolites could be used to identify different species and their abundance in the oral cavity. This could help to assess the severity of the oral infection and aid in the treatment of periodontal disease and dental abscesses. Patients themselves could also use a simple breath test to evaluate the need for seeking treatment, which could prevent severe complications. In general, volatile biomarkers could aid in the identification of different bacterial species, assessment of bacterial growth, and detection of bacterial infections both in vitro and in vivo.

In an earlier study^6^, we used proton-transfer-reaction time-of-flight mass spectrometry (PTR-TOF-MS) to study the time-dependent VOC production profiles of several pathogenic oral bacterial species. The studied anaerobic bacteria have been identified as some of the main culprits in the development of chronic periodontitis and include *Porphyromonas gingivalis*, *Prevotella intermedia*, *Prevotella nigrescence* and *Tannerella forsythia*^2^. All of these bacterial species have also been connected to acute dental abscess^3^. In the earlier study^6^, we found several promising volatile metabolites. However, the analysis method used did not allow an unambiguous identification of all the measured VOCs. In the current study, we aimed to confirm the previously tentatively identified compounds and further investigate the volatile fingerprints of the oral bacteria by gas chromatography–mass spectrometry (GC–MS). Our main goal was to investigate whether pathogenic oral bacteria can be distinguished by their volatile fingerprints, and how this information could be used in clinical diagnostics and microbiological work.

In the second part of this study, we examined the volatile compounds produced by the same pathogenic oral bacteria using PTR-MS. The advantage of PTR-MS compared to the traditionally used GC–MS, is the possibility for extremely sensitive, online measurements. This enables the continuous monitoring of bacterial volatiles and reveals real-time information about the changes in volatile emissions during the bacterial lifecycle. The PTR-MS method uses proton-transfer ionization, which means that the level of fragmentation of sample molecules is small compared e.g. to GC–MS. However, some compounds, such as alcohols, aldehydes and esters, are prone to fragment even with soft ionization^7–15^. For complex samples, such as bacterial headspace or exhaled breath, it is essential that the protonated molecular ion peak is clearly present in the spectrum, and fragmentation is minimized. Otherwise, the identification of the parent compound becomes difficult. Working at lower reduced electric field (*E/N*) values has been shown to significantly reduce the fragmentation of some compounds in PTR-MS^7–15^. In the current study, we examined the effects of different *E/N* conditions on the bacterial headspace measurements to address the problem of fragmentation in complex volatile samples. Our main goal is to determine the ideal *E/N* conditions for bacterial headspace measurements for reliable online analysis.

## Materials and methods

### Bacterial strains and culture methods

Bacterial strains used in this study were: *P. gingivalis* ATCC 33277 (serotype Pg a), *P. gingivalis* ATCC 53978 (W50, serotype Pg b), *P. gingivalis* OMG 434 (serotype Pg c), *P. intermedia* ATCC 25611, *P. nigrescens* ATCC 35563, and *T. forsythia* ATCC 43037. All bacterial strains were acquired from the American Type Culture Collection (ATCC), except for *P. gingivalis* OMG 434, which was from the Gothenburg Culture Collection.

The culture method used has been described in our previous study^6^. All the strains were stored at − 80 °C in frozen skim milk. Strains were activated by streaking onto the Brucella blood agar (BBLTM, 211086) plates, supplemented with horse blood (5% v/v), hemin (5 mg l^−1^) and vitamin K1, except *T. forsythia*, which was cultured on tryptic soy agar (TSA), with n-acetylmuramic acid (NAM) and sheep blood. All bacterial strains were incubated in anaerobic gas mixture (5% CO_2_, 10% H_2_ and 85% N_2_) at 37 °C for 72–120 h. After the incubation, 3.0 ml of phosphate-buffered saline (PBS) was pipetted onto the agar plate, bacteria were gently scraped from the agar, and transferred into a Falcon™ tube. This bacterial suspension was homogenized by gently pipetting. The initial amount of bacteria in the suspensions used ranged between 1.0 and 4.0 × 10^7^ colony forming units (CFUs) per ml. From the 3.0 ml of bacterial suspension, 0.250 ml was pipetted onto a new agar plate, which was placed in an airtight headspace measurement container. Triplicate of each bacterial strain were prepared.

### Bacterial headspace measurements

**Figure 1:**
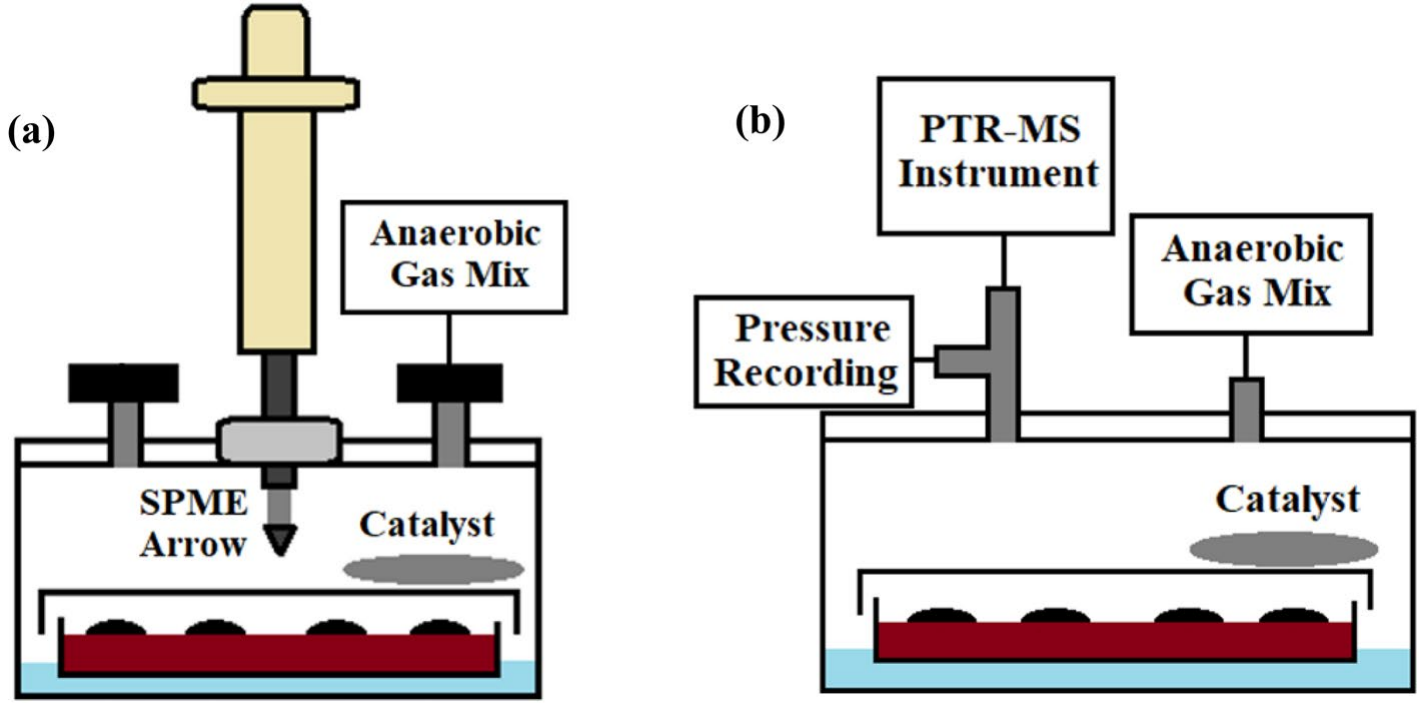
(**a**,**b**) Schematic representation of the bacterial container designed for bacterial headspace measurements. With anaerobic bacteria, excess oxygen is removed with a palladium catalyst. Distilled, sterilized water at the bottom of the container is used to humidify the headspace and prevent bacterial cultures from drying. (**a**) For the GC–MS measurements, the SPME Arrow is injected through a septum. The inlet and outlet are simple on/off two-way valves. (**b**) For the PTR-MS measurement, a continuous gas flow is passed through the container. The flow is controlled with an external mass flow-controller and electronic valves driven by a computer. The same setup is used for reference measurements, with the bacterial culture replaced with an empty petri dish containing a drop of the pure reference compound. Triplicates of each sample were measured.

Figure 1a,b shows the custom-build bacterial containers for the agar-based culture setup, previously described in detail^6^. These setups are used in the current study for (a) the offline GC–MS and (b) the online PTR-MS headspace measurements. The sampling for the GC–MS measurements is described in detail in the next chapter. The full sampling system used for the online PTR-MS headspace measurements has been previously reported in detail^6^. Culture condition are kept at 37 °C and atmospheric pressure during the measurement.

#### GC–MS measurements for volatile identification

Different GC–MS methods have been previously used in microbial VOC analyses^16^. In this study, a GC-instrument (Agilent 6890A) combined to a quadrupole mass spectrometer (Agilent 5973N MSD) using electron ionization (EI) was used for all offline bacterial headspace measurements for the VOC identification. The column used was 30 m long and had an inner diameter of 0.25 mm, with a film thickness of 0.15 µm (DB-1701, J&W Scientific). Separation was achieved by the following temperature program: initial 40 °C with a 2 min hold, ramped at 10 °C/min to 250 °C (5 min hold). A split-splitless injection port was held at 240 °C. Splitless injection was used, with splitless time of 1.0 to 3.0 min from injection. Helium (99.996% from Linde Gas, Espoo, Finland) was used as a carrier gas at a constant flow rate of 1.0 ml/min. The MS operation parameters were as follows: mass scan range of 20–300 u, ion source temperature of 230 °C, quadrupole temperature of 150 °C, ionization energy of 70 eV, and GC–MS transfer line temperature of 250 °C.

**Figure 2:**
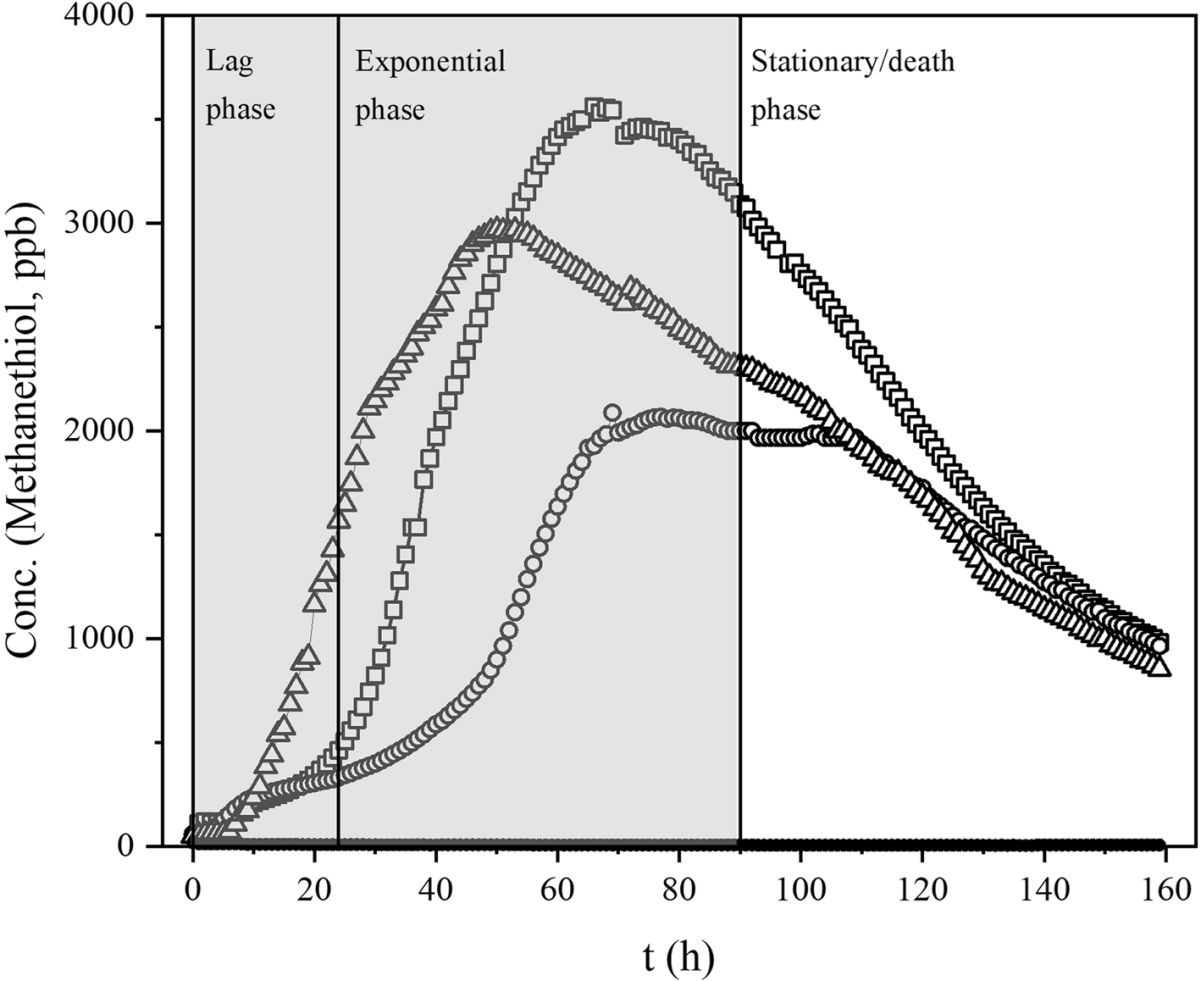
The GC–MS sampling time-points of bacterial headspace. Time-points of 0, 24 and 90 h approximately represent three different phases in bacterial growth on agar: the lag, the exponential growth, and the stationary/death phase, respectively. At the first time-point, the number of compounds and the concentrations produced are assumed to be small and, therefore, most of the volatiles come from the nutrient agar. At the second time-point, the bacteria are growing exponentially, and the production of volatile metabolites accelerates. The last time-point represents a phase, where the available nutrients are running low or are depleted, and bacteria can no longer effectively multiply. Continuous production of methanethiol measured with PTR-MS^6^ is shown here as an indicator of the different phases of bacterial growth.

Volatiles from the headspace of the bacterial cultures were gathered via a polydimethylsiloxane/divinylbenzene solid phase microextraction (SPME) Arrow (1.1 mm outer diameter, 120 µm phase thickness, CTC Analytics AG, Zwingen, Switzerland). The SPME Arrow was directed into the bacterial container via a septum. The SPME Arrow was revealed for 2 min, during which the compounds in the headspace were concentrated on the sorbent. After this, the SPME Arrow was concealed, removed from the container, and immediately introduced to the GC-instrument injection port for 2 min for desorption. The GC–MS measurement protocol was simultaneously initiated. At least two empty runs with the SPME Arrow were done in between every bacterial measurement to avoid contamination from the previous measurement. Triplicate measurements were taken from each bacterial culture and empty nutrient plates at 0, 24 and 90 h, as described in Fig. 2. Empty nutrient plates were used as black control samples.

National Institute of Standards and Technology NIST14 Mass Spectral Library and Analysis Tools were used for the identification of compounds from the measured mass spectra. Pure reference samples of some of the compounds were also measured for further confirmation. Pure reference compounds were obtained from Sigma Aldrich.

#### PTR-TOF-MS measurements for fragmentation analysis

The on-line PTR-MS method has been previously used to investigate the headspace of different bacterial cultures, for example in Refs.^17–21^. The PTR-TOF-MS sampling and analysis methodology used in this study, has also been previously reported in detail^6^. A commercially available PTR-TOF-MS instrument (PTR-TOF 1000, Ionicon) was used for all on-line measurements of bacterial volatiles. The specified mass resolution of the instrument is 1500 m*/Δm* (full-width at half-maximum). In this study, H_3_O^+^ was used as a reagent ion. A separate sensor for CO_2_ (Vaisala, GMP251) was also used.

The operating conditions used have been described in our previous study^6^. Measurements were performed from the mass-to-charge ratio (*m/z*) 17–239 u. PTR-TOF-MS operating conditions were as follows: reduced electric field (*E/N*) of 139 to 81 Td (corresponding to drift tube voltages from 600 to 350 V); drift tube pressure of 2.20 mbar; H_2_O flow of 5.0 standard cubic centimetres per minute (sccm); ion source current of 3.0 mA; inlet flow of 45 sccm (base flow of the instrument). Drift tube and inlet temperatures were kept at 70 °C. Sampling frequency was 1 Hz, i.e. one spectrum was recorded every second. The bacterial headspace samples are saturated with water. To emulate this, a water bath was used with reference samples, as described in Fig. 1b. This resulted in higher humidity in the drift tube than achieved during a normal operation.

The strength of the reduced electric field in the drift tube is expressed as the *E/N* parameter (the ratio of the electric field *E* to the total number density *N* in the drift tube), which can be adjusted by changing the drift tube voltage. Lowering the *E/N* value reduces the drift velocity of the ions in the drift tube resulting in decreased collisional energy, and thus, less fragmentation. The *E/N* values used in this study range from 139 to 81 Td, corresponding to an average center-of-mass kinetic energy range of 30–13 kJ/mol, calculated according to Refs.^22,23^. Lowering the *E/N* value may affect the sensitivity of detection if the increasing protonated water clusters in the drift tube do not react with the reagent neutrals. The H_3_O^+^(H_2_O)_n_ (n = 1 and 2) cluster will react with a neutral only, if the proton affinity of the associated water cluster (H_2_O)_n+1_ is less than that of the neutral, which is often not the case for many volatiles. The H_3_O^+^(H_2_O)_n_ cluster ion formation, induced by a sequential association of H_3_O^+^ with water molecules, is usually prevented by using a sufficiently high *E/N* value leading to collision induced dissociation. However, high *E/N* values also decrease the reaction time in the drift tube, which leads to a smaller conversion efficiency of the primary H_3_O^+^ ion into products. This in turn decreases the sensitivity. Using a high *E/N* value may also increase the degree of fragmentation, which is not desirable especially in complex chemical samples, such as the bacterial headspace or exhaled breath. Protonated water cluster formation is also strongly dependent on the humidity in the drift tube. In our study, the humidity of both bacterial and reference samples is near saturation. For *E/N* values above 110 Td it has been shown that under 100% relative humidity conditions most of the reagent ions are H_3_O^+^ ions. However, at 100 Td, the fraction of H_3_O^+^ ions is already below 50%, and at 90 Td, the fraction is below 10%^22^. This is similar to what we have observed in our measurements (Supplementary Information, p. 8). Balancing the different factors affecting the sensitivity of PTR-MS can be challenging, especially with complex, high humidity samples.

In our study, we decreased the *E/N* value in 2-min steps by adjusting the drift tube voltage. The measurement series was as follows: 600 V (139 Td), 550 V (128 Td), 500 V (116 Td), 450 V (104 Td), 400 V (93 Td) and 350 V (81 Td). During this measurement series, reference substance headspace or bacterial headspace from a container containing the sample, was directed to the PTR-MS instrument for 6 × 2 min. A continuous gas flow of 50 ml/min was directed through the container. Before starting the measurement series, the concentrations in the headspace were allowed to reach a steady state.

The raw PTR-MS signals are normalized to the H_3_O^+^ reagent ion signal of 1000 to aid the comparison of our results to other publications^7^. For the relative abundances, the PTR-MS signal (M + 1) of the protonated molecular ion and the possible fragment ion signals are normalized with respect to the most abundant ion^7,8,12,13^.

## Results

### Identification of bacterial volatiles with GC–MS

**Figure 3:**
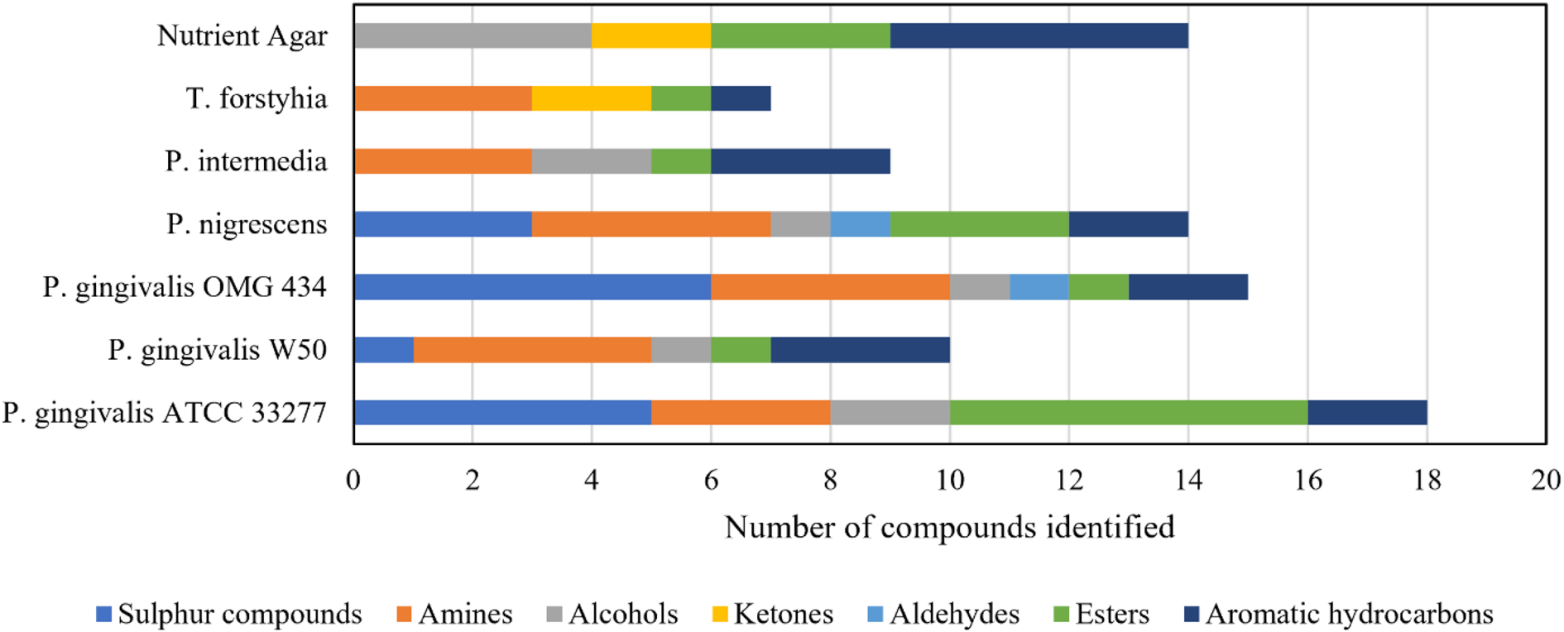
Volatile fingerprints of the studied oral bacteria by compound group. Some of the compounds produced by the bacteria are also produced by the nutrient agar, and for clarity, these compounds are excluded from this figure. Only compounds produced solely by the bacteria are presented.

Table 1 compiles all identified compounds from the offline GC–MS measurements at 0, 24 and 90 h of culturing. Figure 3 presents the volatile fingerprints of the studied bacteria according to the compound group produced. Individual chromatograms for each bacterial species and the nutrient agar are included as Supplementary Data (Supplementary Information, p. 5–7).

**Table 1:** Compounds measured and identified from the headspace of the studied oral bacteria by GC–MS.

Identified signal (bacteria)	Mass (u)	Retention time (min)	*P. gingivalis* ATCC 33277			*P. gingivalis* W50			*P. gingivalis* OMG 434			*P. nigrescens* ATCC 35563			*P. intermedia* ATCC 25611			*T. forsythia* ATCC 43037		
			0 h	24 h	90 h	0 h	24 h	90 h	0 h	24 h	90 h	0 h	24 h	90 h	0 h	24 h	90 h	0 h	24 h	90 h
Methanethiol	48	1.90–2.01	**−**	**+**	**+**	**−**	**−**	**+**	**−**	**−**	**+**	**−**	**−**	**−**	**−**	**−**	**−**	**−**	**−**	**−**
3-Methylbutanal	86	3.10	**−**	**−**	**−**	**−**	**−**	**−**	**−**	**−**	**+**	**−**	**−**	**++**	**−**	**−**	**−**	**−**	**−**	**−**
3-Methyl-2-butanone	86	3.20	**−**	**−**	**−**	**−**	**−**	**−**	**−**	**−**	**−**	**−**	**−**	**−**	**−**	**−**	**−**	**−**	**−**	**+**
2-Methyl-1-propanethiol	90	2.77 3.40	**−**	**+**	**+**	**−**	**−**	**−**	**−**	**−**	**−**	**−**	**−**	**−**	**−**	**−**	**−**	**−**	**−**	**−**
Dimethyl disulfide	94	3.90–3.99	**−**	**+**	**+**	**−**	**−**	**−**	**−**	**−**	**++**	**−**	**−**	**++**	**−**	**−**	**−**	**−**	**−**	**−**
3-Methyl-2-pentanone	100	4.33	**−**	**−**	**−**	**−**	**−**	**−**	**−**	**−**	**−**	**−**	**−**	**−**	**−**	**−**	**−**	**−**	**+**	**+++**
4-Methyl-1-pentanol	102	3.50	**−**	**+**	**+**	**−**	**−**	**−**	**−**	**+**	**+**	**−**	**+**	**++**	**−**	**+**	**−**	**−**	**−**	**−**
Methyl 2-methylpropanoate	102	3.15–3.20	**−**	**+**	**+**	**−**	**++**	**++**	**−**	**++**	**++**	**−**	**+**	**++**	**−**	**+**	**++**	**−**	**+**	**−**
Indole	117	14.46–14.49	**−**	**+**	**++**	**−**	**+**	**+++**	**−**	**++**	**++**	**−**	**+**	**+++**	**−**	**+**	**++**	**−**	**++**	**++**
*S*-Methyl butanethioate	118	6.10	**−**	**−**	**−**	**−**	**−**	**−**	**−**	**−**	**++**	**−**	**−**	**++**	**−**	**−**	**−**	**−**	**−**	**−**
1-Ethyl-4-methylbenzene	120	6.36	**−**	**−**	**+**	**−**	**−**	**+**	**−**	**−**	**+**	**−**	**−**	**+**	**−**	**+**	**+**	**−**	**−**	**+**
1,3,5-Trimethylbenzene	120	7.60	**−**	**−**	**+**	**−**	**−**	**+**	**−**	**−**	**+**	**−**	**−**	**+**	**−**	**−**	**+**	**−**	**−**	**−**
Dimethyl trisulfide	126	7.51–7.53	**−**	**−**	**++**	**−**	**−**	**−**	**−**	**+**	**+++**	**−**	**−**	**++**	**−**	**−**	**−**	**−**	**−**	**−**
Propylcyclohexane	126	5.83	**−**	**−**	**−**	**−**	**−**	**−**	**−**	**−**	**++**	**−**	**−**	**+**	**−**	**+**	**−**	**−**	**−**	**−**
Ethyl 2-methylbutanoate	130	5.21	**−**	**−**	**++**	**−**	**−**	**−**	**−**	**−**	**−**	**−**	**−**	**+++**	**−**	**−**	**−**	**−**	**−**	**−**
2-Ethyl-1-hexanol	130	8.90	**−**	**−**	**−**	**−**	**−**	**−**	**−**	**−**	**−**	**−**	**−**	**−**	**−**	**−**	**++**	**−**	**−**	**−**
3-Methyl-1-butanol acetate	130	5.87	**−**	**+**	**++**	**−**	**−**	**−**	**−**	**−**	**−**	**−**	**−**	**−**	**−**	**−**	**−**	**−**	**−**	**−**
*S*-Methyl-3-methylbutanethioate	132	6.86	**−**	**++**	**++**	**−**	**−**	**−**	**−**	**−**	**+++**	**−**	**−**	**++**	**−**	**−**	**−**	**−**	**−**	**−**
1-Methyl-2-propan-2-ylbenzene	134	7.94	**−**	**−**	**−**	**−**	**+**	**+**	**−**	**−**	**−**	**−**	**−**	**−**	**−**	**−**	**+**	**−**	**−**	**−**
3-Methyl-1-butyl propanoate	144	7.30	**−**	**+**	**++**	**−**	**−**	**−**	**−**	**−**	**−**	**−**	**−**	**−**	**−**	**−**	**−**	**−**	**−**	**−**
1-Ethylindole	145	13.75	**−**	**+**	**+**	**−**	**+**	**++**	**−**	**−**	**+**	**−**	**+**	**+**	**−**	**+**	**+**	**−**	**+**	**−**
1-Methyl-1,2,3,4-tetrahydroisoquinoline	147	12.53	**−**	**−**	**−**	**−**	**−**	**++**	**−**	**−**	**+**	**−**	**−**	**+**	**−**	**−**	**−**	**−**	**−**	**−**
Dimethyl tetrasulfide	158	11.60	**−**	**−**	**−**	**−**	**−**	**−**	**−**	**−**	**+**	**−**	**−**	**−**	**−**	**−**	**−**	**−**	**−**	**−**
3-Methylbutyl 2-methylpropanoate	158	7.90	**−**	**−**	**+**	**−**	**−**	**−**	**−**	**−**	**−**	**−**	**−**	**−**	**−**	**−**	**−**	**−**	**−**	**−**
**Identified signal (bacteria + agar)**																				
Ethanol	46	2.10–2.20	**−**	**+**	**+**	**+**	**+**	**++**	**−**	**+**	**++**	**+**	**+**	**++**	**+**	**++**	**+++**	**−**	**−**	**−**
Acetone	58	2.31	**+**	**+**	**+**	**+**	**+**	**+**	**+**	**+**	**++**	**+**	**+**	**++**	**+**	**+**	**+**	**+**	**++**	**++**
3-Methyl-1-butanol	88	4.37–4.44	**+++**	**++++**	**+++**	**+++**	**+++**	**+++**	**+++**	**+++**	**+++**	**+++**	**+++**	**+++**	**++**	**+++**	**++**	**+++**	**+++**	**+++**
Toluene	92	4.02–4.10	**++**	**+**	**+**	**++**	**+++**	**+++**	**++**	**+**	**++**	**+**	**++**	**++**	**++**	**+++**	**+++**	**++**	**+++**	**+++**
Methyl cyclohexane	98	3.08	**+**	**+**	**+**	**+**	**+**	**+**	**+**	**+**	**++**	**+**	**+**	**++**	**+**	**+++**	**+++**	**−**	**−**	**−**
Methyl 2-methylprop-2-enoate	100	3.45–3.50	**+**	**+**	**++**	**+**	**+**	**+**	**+**	**+**	**++**	**+**	**+**	**−**	**+**	**++**	**++**	**++**	**++**	**+++**
3-Methyl-3-pentanol	102	8.45	**++**	**+**	**−**	**++**	**−**	**−**	**+++**	**−**	**−**	**+++**	**+**	**−**	**++**	**−**	**−**	**−**	**−**	**−**
Ethynylbenzene	102	6.03	**−**	**−**	**−**	**++**	**−**	**−**	**++**	**+**	**+**	**++**	**−**	**−**	**+**	**−**	**−**	**−**	**−**	**−**
Styrene	104	6.09–6.14	**++**	**++**	**++**	**++**	**++**	**++**	**++**	**++**	**++**	** + **	**++**	**++**	**+**	**+**	**++**	**+**	**+**	**+++**
Ethylbenzene	106	5.40, 6.01	**+++**	**+++**	**++**	**+++**	**+++**	**+++**	**++**	**+++**	**+++**	**+**	**+++**	**+++**	**+++**	**+++**	**+++**	**++**	**+++**	**+++**
Xylene	106	5.39–5.40 5.85–6.10	**+**	**+**	**++**	**+**	**+**	**+**	**+**	**+**	**++**	**+**	**++**	**++**	**−**	**+**	**++**	**+**	**++**	**+++**
Ethyl cyclohexane	112	4.30	**++**	**++**	**+++**	**+++**	**+++**	**+++**	**+++**	**+++**	**+++**	**+++**	**+++**	**+++**	**++**	**+++**	**++ + **	**−**	**−**	**+**
Ethyl butanoate	116	4.64	**++**	**++**	**+++**	**−**	**−**	**−**	**−**	**−**	**++**	**−**	**−**	**++**	**−**	**−**	**−**	**−**	**−**	**−**
5-Methyl-2-heptanone	128	7.74	**+**	**+**	**++**	**−**	**−**	**−**	**−**	**−**	**−**	**−**	**−**	**−**	**−**	**−**	**−**	**−**	**−**	**++**
6-Methyl-1-heptanol	130	8.90	**+**	**+**	**−**	**+**	**+**	**−**	**−**	**−**	**−**	**+**	**+**	**−**	**+**	**−**	**−**	**−**	**−**	**−**
3-Methylbutyl 3-methylbutanoate	172	9.36	**+**	**++**	**++**	**++**	**+**	**++**	**++**	**+**	**+**	**++**	**+**	**+**	**+**	**+**	**−**	**−**	**−**	**−**

The first part of the table presents compounds solely produced by the bacteria. The latter part of the table presents compounds produced by the nutrient agar and bacteria. Markings indicate the relative signal intensity in the chromatograms (“−“ = no signal, “+” = small signal, “++” = moderate signal, “+++” = large signal). Retention times refer to chromatograms presented in the Supplementary Data of this article.

Compounds produced by all bacteria studied include: Methyl 2-methylpropanoate (methyl isobutyrate, M = 102 u), indole (M = 118 u), 1-ethyl-4-methylbenzene (1-ethyl toluene, M = 120 u), 1,3,5-trimethylbenzene (mesitylene, M = 120 u), and 1-ethylindole (M = 145 u).

Compounds produced by several of the studied bacteria include: Methanethiol (M = 48 u), 3-methylbutanal (M = 86 u), dimethyl disulfide (DMDS, M = 94 u), 4-methyl-1-pentanol (M = 102 u), *S*-methyl butanethioate (*S*-methyl thiobutyrate, M = 118 u), dimethyl trisulfide (DMTS, M = 126 u), propylcyclohexane (M = 126 u), ethyl (2*S*)-2-methylbutanoate (ethyl 2-methylbutyrate, M = 130 u), *S*-methyl-3-methylbutanethioate (methanethiol isovalerate; M = 132 u), 1-methyl-2-propan-2-ylbenzene (o-cymene, M = 134 u), and 1-methyl-1,2,3,4-tetrahydroisoquinoline (1MeTIQ, M = 147 u).

Compounds that were produced only by a certain bacterial species or strain include: 3-methyl-2-butanone (M = 86 u, Tf), 2-methyl-1-propanethiol (M = 90 u, Pg a), 3-methyl-2-pentanone (M = 100 u, Tf), 2-ethyl-1-hexanol (M = 130 u, Pi), 3-methyl-1-butanol acetate (isoamyl acetate, M = 130 u, Pg a), 3-methyl-1-butyl propanoate (isoamyl propionate, M = 144 u, Pg a), 3-methylbutyl 2-methylpropanoate (isoamyl isobutyrate, M = 158 u, Pg a) and dimethyl tetrasulfide (M = 158 u, Pg c).

Some of the compounds listed were also found and tentatively identified in our earlier study, where we measured the same bacteria using PTR-MS^6^. Comparison of the earlier results to the confirmed GC–MS signals of the current study are summarized in Table 2.

**Table 2:** Comparison of previously tentatively identified PTR-MS signals^6^ to the results obtained in this study using GC–MS and the fragmentation pattern analysis of the PTR-MS signals.

PTR-MS signal (M + 1) (u)	Tentative identification (PTR-MS)^6^	Confirmed signals (this study)
35	Hydrogen sulphide	Hydrogen sulphide (contribution also from 2-methyl-propanethiol and some other fragment ions)
49	Methanethiol	Methanethiol (contribution also from DMDS and DMTS fragment ions)
57	Butene, acrolein	Combination of fragment ions from: 3-methyl-2-pentanone, 5-methyl-2-heptanone (agar), 2-methyl-1-propanethiol
59	Acetone	Acetone, combination of fragment ions: 3-methyl-2-pentanone, 5-methyl-2-heptanone (agar)
63	DMS	Combination of fragment ions
69	Isoprene, fragment ion (C_5_H_9_^+^)	Combination of fragment ions from: 3-methylbutanal, 5-methyl-2-heptanone (agar)
71	Pentene/isopentene, crotonaldehyde	Combination of fragment ions from: isoamyl propionate, ethyl isobutyrate, 3-methyl-1-butanol (agar)
85	Cyclopentanone	Combination of fragment ions from: 4-methyl-1-pentanol
87	Pentanone/methyl butanal, diacetyl, methyl butenol	3-Methyl-1-butanal
91	Butanethiol, methylthiourea, tropylium ion/other fragment ion (C_7_H_7_^+^)	2-Methyl-1-propanethiol
95	DMDS, phenol, fragment ion (C_7_H_11_^+^)	DMDS
101	Hexanal, acetyl acetone, MIBK	3-Methyl-2-pentanone, combination of fragment ions from: 5-methyl-2-heptanone (agar), 4-methyl-1-pentanol, methyl metacrylate (agar), methyl isobutyrate
118	Indole, benzyl cyanide	Indole

In addition to the compounds mentioned above, we identified several compounds that are produced both by the bacteria and the nutrient agar, and sometimes in similar amounts. There are also several compounds that are produced only by the nutrient agar. Some of the compounds produced by the agar seem to be depleted by the bacteria, or otherwise, during the culturing. As we are mostly interested in the compounds clearly produced by the bacteria, the compounds originating from other sources are not discussed further in this article. These compounds include ethanol (M = 46 u), acetone (M = 58 u), 3-methyl-1-butanol (M = 88 u), toluene (M = 92 u), methyl cyclohexane (M = 98 u), methyl 2-methylprop-2-enoate (methyl methacrylate, M = 100 u), 3-methyl-3-pentanol (M = 102 u), ethynylbenzene (M = 102 u), styrene (M = 104 u), xylene (M = 106), ethyl benzene (M = 106 u), ethyl cyclohexane (M = 112 u), ethyl butanoate (M = 116 u), 5-methyl-2-heptanone (M = 128 u), 6-methyl-1-heptanol (M = 130 u), and 3-methylbutyl 3-methylbutanoate (M = 172 u). Some GC–MS peaks were found to originate from polysiloxane in the GC column or divinylbenzene and polysiloxane in the SPME Arrow. Masses of these peaks included 73, 77, 107, 142, 151, 207 and 281 u. Additionally, several solvents were also found, such as propanol (M = 60 u), benzaldehyde (M = 106 u), benzyl alcohol (M = 108 u), and methyl heptanone (M = 126 u).

### Effect of *E/N* value on the PTR-MS fragmentation

Most of the PTR-MS signals (M + 1) increase or remain relatively unchanged as the *E/N* value is decreased from 139 to 81 Td, which is to be expected with less fragmentation. However, several signals decrease with decreased *E/N* value, which might indicate that these signals are fragment ions. As the parent molecules fragment less, these signals decrease or disappear. These signals include: 41, 42, 43, 44, 45, 46, 57, 59, 61, 69, 71, 73, 75, 79, and 80 u. All of these masses are connected to important fragment ions of alcohols, aldehydes, ketones, carboxylic acids and esters.

**Figure 4:**
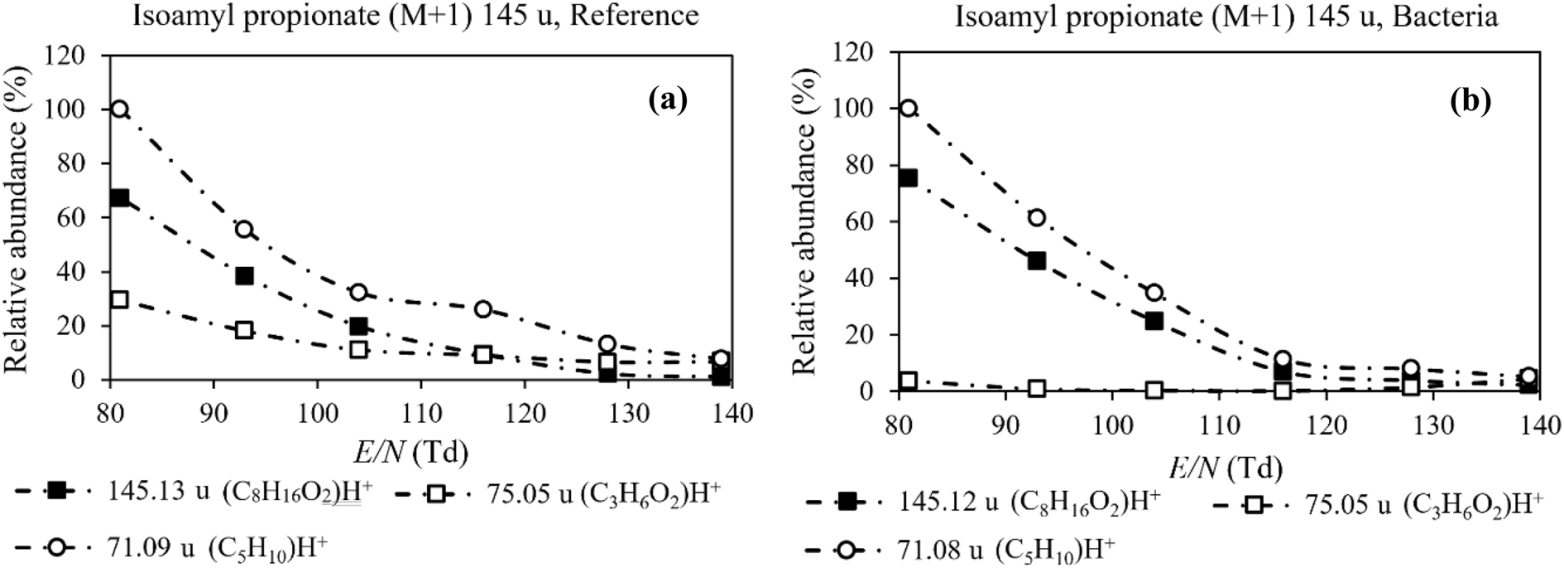
(**a**,**b**) Relative abundances of the PTR-MS signals (M + 1) for the protonated molecular ion and the most important fragments of isoamyl propionate. Additional diagrams with elemental compositions and accurate masses can be found as Supplementary Data of this article. Signals are normalized to the most abundant signal (100%) in any of the different *E/N* conditions.

Relative abundances of compounds identified in this study, and their possible fragment ions measured with PTR-MS, are presented in the Supplementary Data (Supplementary Information, p. 1–3). Measurements from pure reference samples and bacterial headspace samples are presented side-by-side. The presented diagrams describe the connection between the protonated molecular ion signal and the possible fragment ions. Figure 4a,b in the article presents results for one of these compounds as an example. The measured accurate masses of different molecular species are compared to their calculated exact masses to confirm the assignments (Supplementary Information p. 4).

## Discussion

### Potential volatile biomarkers identified from the bacterial headspace with GC–MS

We found clear differences in the volatile fingerprints of the studied oral bacteria. Large differences were discovered especially in volatile sulphur compound and ester production. Amines, alcohols and aromatic hydrocarbons were produced in similar, large amounts by most of the studied bacteria. Volatile aldehyde and ketone production was rare and seemed more specific to certain species. Differences were also found between the different strains of *P. gingivalis*. It should be noted that the nutrient agar did not produce any of the identified sulphur compounds, amines or aldehydes. In contrast, the nutrient agar did produce alcohols, ketones and hydrocarbons in abundance.

According to literature, methanethiol is one of the most abundant compounds produced by *P. gingivalis*, *P. intermedia* and other pathogenic oral bacteria^5^. We have also previously measured real-time methanethiol production from oral bacteria with PTR-MS^6^. Our current GC–MS study from the same bacteria confirms these findings. These results also confirm our previous suggestion that *P. gingivalis* produces methanethiol in large amounts, and that it is one of the most important dividers between *P. gingivalis* and the other tested oral pathogens. More efforts should be made in the future to evaluate if and how methanethiol could be used as an indicator for *P. gingivalis* in (1) in vitro culturing or (2) in vivo measurements, for example, from human exhaled breath or saliva.

We also identified several other sulphur compounds from the headspace of the studied oral bacteria. All *P. gingivalis* strains produce these compounds. In addition, *P. nigrescens* produces some of these compounds, but not all. No sulphur-containing compounds were identified from the headspace of *P. intermedia* and *T. forsythia*. Sulphur compounds could be a promising way to distinguish *P. gingivalis* from the other oral bacteria. Instead of using just one sulphur compound, efficient identification could be achieved by using a volatile fingerprint of multiple sulphur compounds. Further studies are needed to examine, whether other members of *Porphyromonas* genus produce sulphur compounds, for example *P. endodontalis*, which plays an important role in endodontic infections^24^.

Four volatile amines were found from the headspace of the oral bacteria. We have previously reported the possible production of volatile indole by the same bacteria^6^. Our current measurements with GC–MS confirm this finding. In the traditional microbiological indole test, *P. gingivalis*, *P. intermedia* and *P. nigrescens* are indole positive species, while *T. forsythia* is an indole negative species. However, our current GC–MS and previous PTR-MS measurements show that *T. forsythia* produces volatile indole. In an earlier study, volatile indole was used as an in vitro marker for *E. coli* cultured on agar and in blood^25^. This prospect should also be investigated for the oral bacteria as an alternative for the traditional indole test in liquid medium. In addition to indole, 1-ethylindole is also produced in very small amounts by all the bacteria.

Another important amine identified in this study is 1-methyl-1,2,3,4-tetrahydroisoquinoline (1MeTIQ), which belongs to tetrahydroisoquinolines (TIQs)—a widespread group of compounds found from bacteria, plants and animals. This compound has also pharmaceutical properties. To our knowledge, bacterial production of 1MeTIQ has not been reported previously. In our current study, we found 1MeTIQ from the headspace of *P. nigrescens* and two *P. gingivalis* strains at the latter part of culturing. They produce it in small, but clearly observable levels. As this is a novel finding, the production capacity, relevance, and biomarker potential of 1MeTIQ by different bacteria has to be investigated in the future.

The studied bacteria produce many different esters. We recorded significant production of methyl isobutyrate from all species. The other identified esters are produced only by *P. gingivalis* (a) and *P. nigrescens*. Interestingly, out of the three *P. gingivalis* strains, only (a) was found to produce esters other than methyl isobutyrate. The combination of these esters could be used as an identifier between the three strains. It would also be interesting to see if some clinical or mutant strains of *P. gingivalis* are able to produce esters or not. Esters are the major distinguisher of *P. gingivalis* (a) from all of the other studied bacteria.

Many aromatic hydrocarbons are also found from the headspace of the studied bacteria. They all produce 4-ethyltoluene and mesitylene in small amounts, except *T. forsythia*, which does not produce mesitylene. In contrast, o-cymene is only found from the headspace of *P. gingivalis* (b) and *P. intermedia*. The biomarker potential of mesitylene and 4-ethyltoluene is limited, because they are produced by most of the studied bacteria. However, they could be used as non-specific markers for oral anaerobes, if other microbes in the oral cavity do not produce them. This could have importance in periodontitis, where the proportion of anaerobes increase due to the formation of pathologically deepened periodontal pockets and anaerobic conditions. Mesitylene has been connected earlier to cariogenic bacteria^26^, but to our knowledge 4-ethyltoluene has not been previously identified as a bacterial VOC. However, the isomeric compound 3-ethyltoluene has been connected to cariogenic bacteria^26^. The remaining aromatic compound, o-cymene, could be a potential biomarker for *P. gingivalis* (b) and *P. intermedia*. It could also be used as one of the separators between the *P. gingivalis* strains with different virulence potential.

We also identified alcohols, ketones and one aldehyde from the headspace of the bacteria. Alcohols, ketones and aldehydes are the main separating compounds between *T. forsythia* and the other bacterial species. Presence of ketones and the absence of alcohols and aldehydes, as well as sulphur compounds, in the headspace, is unique for *T. forsythia*. In addition, the nutrient agar produces large amounts of alcohols and ketones. In fact, the studied bacteria produce less alcohols and ketones than the nutrient agar, which limits the use of these compounds as volatile biomarkers in vitro for these bacteria. Aldehydes are the rarest compound group produced. Only 3-methylbutanal is found from the headspace of *P. nigrescens* and *P. gingivalis* (c). Rarity makes aldehydes promising as possible volatile biomarkers. For example, 3-methylbutanal could be used to distinguish between the different strains of *P. gingivalis*.

Most of the compounds identified in this study have also been found from the human exhaled breath^27^. Some compounds not found from the exhaled breath, have been observed in either saliva or faeces instead^27^. Compounds not found from human secretions include: 1MeTIQ, 3-methylbutyl 2-methylpropanoate, 1-methyl-2-propan-2-ylbenzene, 3-methyl-1-butyl propanoate, 1-ethylindole, and dimethyl tetrasulfide.

### PTR-MS fragmentation patterns of bacterial VOCs

Earlier studies show that many compounds fragment in the drift tube of the PTR-MS instrument, when using high reduced electric field values^7–15,28–31^. Compounds especially prone to fragmentation include alcohols, aldehydes, carboxylic acids and esters. Many of the compounds produced by the bacteria studied for this article can be assumed not to fragment significantly, because of their small size or the lack of branching or longer substituent groups. The presence of the protonated molecular ion signals for these compounds further suggests that they do not fragment significantly in our PTR-MS system. However, several produced compounds can fragment considerably, and for those, PTR-MS analysis could benefit from lower *E/N* conditions.

In general, the reference sample measurements for the fragmenting compounds (Supplementary Information, p. 1–3) show clear reduction or absence of the protonated molecular ion signal at high *E/N* values. In addition, high fragment ion signals can be observed for many of these compounds. Same trends can be observed for the bacterial measurements (Supplementary Information, p. 1–3). However, the comparison between the pure reference sample and bacterial measurements is not straightforward, because of the complexity of the bacterial headspace. Many different compounds can fragment and produce the same ions. Thus, it can be challenging to distinguish the origin of the contributions to fragment ion signals. Consequently, the fragmentation of specific compounds in the PTR-MS should be evaluated primarily with the help of the reference measurements. The fragmentation trends can then be further used as guidelines for the analysis of bacterial samples. The molecular ion signals and fragment ions were assigned by comparing the measured accurate mass to the calculated exact mass of the molecular species (Supplementary Information, p. 4). Most reference sample mass differences were below 0.01 u, except for three signals. These are discussed below, where relevant. Bacterial sample mass differences are also mostly below 0.01 u. However, several signals have a larger mass difference in the range of 0.05–0.10 u, which can hinder signal assignment. Bacterial PTR-MS findings are confirmed in this study with GC–MS, however, so any difficulties in the signal assignment are compensated.

It is clear from the results of pure reference samples (Supplementary Information, p. 1) that for the measured alcohols and aldehydes, fragmentation has a significant effect on the spectra of these compounds. For 4-methyl-1-pentanol (C_6_H_14_O), 2-ethyl-1-hexanol (C_8_H_18_O) and 3-methylbutanal (C_5_H_10_O), the protonated molecular ion signal [(M + 1) = 103.11, 131.14 and 87.08 u, respectively] is negligible. This finding is in agreement with previous PTR-MS studies that have demonstrated that for alcohols and aldehydes the protonated molecular ion is usually not the most abundant signal in the spectra and can be missing altogether, especially when using H_3_O^+^ as a reagent ion^8,12,13,28^. The dehydration product of these alcohols and aldehydes (C_6_H_12_, C_8_H_16_ and C_5_H_10_, respectively) is usually the more abundant signal, with plethora of smaller fragment ions also present, especially in high *E/N* conditions. Our reference measurements show a clear increase in both the protonated molecular ion and dehydration product [(M + 1) = 85.10, 113.13 and 71.09 u, respectively] signals, with a decreasing *E/N* value, while the signals for the small fragment ions, such as 41.03 u (C_3_H_5_^+^), decrease drastically. According to our findings, low to moderate *E/N* conditions (90–110 Td) should be used for both alcohols and aldehydes to prevent fragmentation and to increase the protonated molecular ion signal. It should also be noted that the dehydration product is the larger signal in all tested *E/N* conditions, and therefore, should be used together with the protonated molecular ion signal for better analysis of alcohols and aldehydes in PTR-MS.

Esters are reported to fragment more readily with increasing carbon chain length ratio (CLR) in high *E/N* conditions (≥ 120 Td)^29^. This ratio is the number of carbon atoms in the alcohol part divided by the number of carbon atoms in the acid part. Esters, with a ratio larger than 0.75, are more likely to undergo hydrolysis. Consequently, for these compounds, the hydrolysis products are abundantly present in the mass spectrum, while the protonated molecular ion signal is small. Esters identified in our current study, with CLR larger than 0.75, are isoamyl propionate (C_8_H_16_O_2_) and isoamyl isobutyrate (C_9_H_18_O_2_). Our PTR-MS results (Supplementary Information, p. 2) show that, for isoamyl isobutyrate, the protonated molecular ion signal [(M + 1) = 159.14 u] is negligible in high *E/N* conditions, and for isoamyl propionate, the signal [(M + 1) = 145.13 u] is only slightly larger. This confirms the earlier reports. For isoamyl propionate (Fig. 4a,b) the major fragment ions are present at masses 75.05 u ((C_3_H_6_O_2_)H^+^) and 71.09 u ((C_5_H_10_)H^+^). In very high *E/N* conditions (> 120 Td), the protonated molecular ion and most of the fragment ion signals are small, but comparable. With decreasing *E/N* value, the signal 71.09 u becomes the largest signal, with the protonated molecular ion trailing it. Other signals become small in comparison to these two in low *E/N* conditions (80–90 Td). Unfortunately, in bacterial measurements, the signal 71.09 u has a major contribution from 3-methyl-1-butanol produced by the nutrient agar. Consequently, this signal is not as useful in the identification of isoamyl propionate from bacterial headspace. For isoamyl isobutyrate, the fragments from the acid ((C_4_H_8_O_2_)H^+^, 89.07 u) and alcohol ((C_5_H_10_)H^+^, 71.09 u) parts of the molecule are small throughout the measurements. Instead, small fragment ions are the most abundant signals. The abundance of these smaller fragment ions decreases rapidly with decreasing *E/N* value, while the protonated molecular ion signal increases. Our results indicate that using low to moderate (90–105 Td) conditions for isoamyl propionate and isoamyl isobutyrate is advised to maximize the protonated molecular ion signals.

According to literature, other compounds identified in this study that are prone to fragment in the drift tube, include o-cymene (C_10_H_14_)^10,30^, DMDS (C_2_H_6_S_2_) and DMTS (C_2_H_6_S_3_)^31^. The results of our current study (Supplementary Information, p. 2–3) show a clear increase in the protonated molecular ion signal ((M + 1) = 135.12, 95.01 and 126.99 u, respectively) with decreasing *E/N* value for all these compounds. However, in contrast to alcohols, aldehydes and esters, the protonated molecular ion signals are clearly visible even in high *E/N* conditions (> 120 Td). They are also one of the largest signals through high (120 Td) to low (80 Td) *E/N* conditions. For o-cymene (Supplementary Information, p. 2), signal 93.09 u ((C_7_H_8_)H^+^) is the largest fragment ion. Earlier studies suggest that this fragment should be large in high *E/N* conditions and small in low conditions^10,11,30^, but our results suggest that the fragment signal follows the trend of the protonated molecular ion. The 93.09 u signal differs from the exact mass of (C_7_H_8_)H^+^ by 0.022 u, which could hinder the assignment of the peak. However, the mass ion signal of (C_10_H_14_)H^+^ is accurate, with a mass difference of 0.001 u, which confirms the presence of cymene. According to literature, DMDS produces fragment ions 79 u (CH_3_S_2_^+^) and 49 u ((CH_4_S)H^+^), and DMTS fragment ions 93 u (CH_3_S_2_CH_2_^+^), 81 u (CH_3_S_2_H_2_^+^), 79 u (CH_3_S_2_^+^), 61 u (CH_3_SCH_2_^+^), 49 u and 45 u (CHS^+^)^31^. In our reference measurements (Supplementary Information, p. 3), the DMDS protonated molecular ion is clearly the largest signal throughout, with the fragment ion signals [(M + 1) = 78.98 and 49.01 u, respectively] comparable to it in high *E/N* conditions, but negligible in moderate to low *E/N* conditions. For DMTS, the protonated molecular ion signal is also observable even in high *E/N* conditions, a fragment ion at 49.01 is the largest signal, and most of the other fragment ions [(M + 1) = 93.00, 80.98, 78.98, 61.02 and 44.98 u] are of similar abundance. The protonated molecular ion signal increases rapidly with decreasing *E/N* value, while the fragment ion signals remain unchanged or increase only moderately. The mass differences for the signals at 78.98 and 126.99 u are 0.014 and 0.015, respectively. This could affect the assignment of the peaks, however, most of the other important fragment ions are accurate. Especially the signals for (CH_4_S)H^+^ (49.01 u) and CH_3_S_2_H_2_^+^ (80.98 u), with mass differences of 0.001 u, indicate the presence of DMDS and DMTS. For o-cymene, DMDS and DMTS, using moderate *E/N* conditions helps to distinguish the protonated molecular ion signal from the fragments, while keeping the formation of water adducts and clusters in the PTR-MS system at acceptable levels. In addition, the identification of o-cymene could be aided by examining both the protonated molecular ion and the 93 u fragment ion signals.

### Limitations of the study

We used the same agar-based culturing setup, as in our earlier study^6^, to reliably compare the results from the two studies. We acknowledge that a broth-based culturing system would be better for bacterial VOC analysis, because it provides the possibility for easy growth-rate determination. In our current agar-based model, measuring the growth-rate would result in losing the bacterial sample, which is undesirable for VOC analysis with multiple measurement time-points. In the next phase of our research, we will reproduce the VOC measurements described in this article with broth cultures, with emphasis on the effects of nutrient composition on the bacterial VOCs.

Another difficulty in bacterial VOC measurements is the complexity of the samples. Bacterial headspace produces dozens of signals in both GC–MS and PTR-MS spectra, which can be hard to interpret, even with the help of mass spectral libraries and reference samples. Humidity of the samples further complicate the analysis. One of our aims in this study, was to analyse the fragmentation patterns of oral bacterial volatiles and provide information about the preferred PTR-MS ionization conditions. However, comprehensive fragmentation analysis is challenging with such complex samples, and the results gained might be limited to the specific measurement setup and conditions.

## Conclusions

The volatile fingerprints of the different bacterial species—*P. gingivalis, P. intermedia, P. nigrescens* and *T. forsythia*—differ significantly. Abundance of sulphur compounds clearly separate *P. gingivalis* and *P. nigrescens* from the other two species, as well as the nutrient agar. The separating volatiles include compounds such as DMDS, DMTS, *S*-methyl-3-methylbutanethioate and *S*-methyl butanethioate. However, *P. gingivalis* produces an even wider variety of sulphur compounds than *P. nigrescens*. Methanethiol, 2-methyl-1-propanethiol and dimethyl tetrasulfide are the separating sulphur compounds between these two bacterial species, with methanethiol providing the clearest separation. A quinoline compound, 1MeTIQ, is also produced only by *P. gingivalis* and *P. nigrescens,* separating them from *P. intermedia* and *T. forsythia.* The similarities between the volatile fingerprints of *P. gingivalis* and *P. nigrescens* makes it difficult to differentiate between them. However, only *P. nigrescens* and *P. gingivalis* (c) produce aldehydes, which could be used to separate the two species.

Alcohols, ketones and aromatic hydrocarbons are produced in large numbers by the bacteria, as well as, the nutrient agar. Consequently, their use as in vitro biomarkers for the studied bacteria could be challenging. However, these compounds could have potential in vivo, when the contribution from the nutrient agar is removed. Their potential, for example, as breath or saliva biomarkers should be investigated further. Amines, on the other hand, are common volatile metabolites for all the studied bacteria, but are mostly absent in the nutrient agar headspace. Their use as markers for bacterial growth should be investigated in the future, especially in the case of indole.

The main differences between the three different *P. gingivalis* strains are: the abundant production of esters by *P. gingivalis* (a), the aldehyde production by *P. gingivalis* (c), and the overall smaller production of volatiles by *P. gingivalis* (b). The volatile fingerprints of avirulent strains (a) and (c) are clearly more alike than that of the virulent strain (b). This could indicate differences in the volatile fingerprints according to virulence.

We conclude that the studied oral bacteria can be separated by their respective volatile fingerprints. In the future they could be used for: (1) in vitro analysis and identification of bacterial cultures, (2) in vivo measurements of surfaces or areas colonized by bacteria, (3) in vivo analysis of exhaled breath to assess periodontal disease or dental abscess.

In our online PTR-MS measurements, we found that alcohols, aldehydes and esters are especially prone to fragmentation in high *E/N* conditions. In reference sample measurements, the protonated molecular ion was negligible or missing from the mass spectra for these compounds. Fragmentation and missing molecular ion signal make spectral analysis cumbersome especially for complex mixtures, such as the bacterial headspace. Using low or moderate *E/N* conditions can decrease the level of fragmentation, and aid in the analysis and identification of compounds with the PTR-MS method.

The combination of PTR-MS and GC–MS, both utilized in this study, is a powerful tool for the analysis of bacterial volatiles. The superior sensitivity and the possibility for continuous, real-time monitoring make PTR-MS especially suitable for analysis of living organisms. On the other hand, GC–MS provides concrete identification of bacterial volatiles, which is often impossible with PTR-MS, especially from complex mixtures.

## Supplementary Information

Supplementary Information.
