# Supplementary material for: Identifying volatile in vitro biomarkers for oral bacteria with proton-transfer-reaction mass spectrometry and gas chromatography–mass spectrometry
Source: Sci Rep. 2021 Aug 19;11:16897. doi: 10.1038/s41598-021-96287-7 (PMC8377122; doi:10.1038/s41598-021-96287-7)
Supplement: Supplementary file 1 — Supplementary Information. [file 41598_2021_96287_MOESM1_ESM.pdf]

SUPPLEMENTARY INFORMATION FOR "Identifying volatile *in vitro* biomarkers for oral bacteria with proton-transfer-reaction mass spectrometry and gas chromatography-mass spectrometry"

K. Roslund, M. Lehto, P. Pussinen, K. Hartonen, P.-H. Groop, L. Halonen and M. Metsälä

TABLE OF CONTENTS

**PTR-MS fragmentation diagrams**	**Page**
4-methyl-1-pentanol	1
2-ethyl-1-hexanol	1
3-methylbutanal	1
Isoamyl propionate (3-methyl-1-butyl propanoate)	2
Isoamyl isobutyrate (Methyl 2-methylpropanoate)	2
o-Cymene (1-methyl-2-propan-2-ylbenzene)	2
Dimethyl disulfide	3
Dimethyl trisulfide	3
**PTR-MS elemental compositions, accurate masses and exact masses**	4
**GC-MS bacterial chromatograms**	
Nutrient agar	5
P. nigrescens	5
T. forsythia	5
P. gingivalis (a) 33277	6
P. gingivalis (b) W50	6
P. gingivalis (c) OMG434	6
P. intermedia	7
**H_3O^+ distribution in the drift tube with different *E/N* values**	8

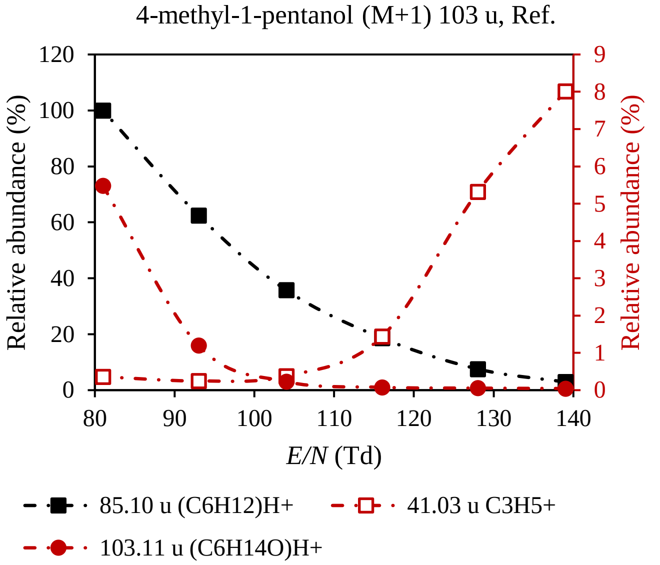
4-methyl-1-pentanol (M+1) 103 u, Ref.
Relative abundance (%)
E/N (Td)
85.10 u (C6H12)H+
41.03 u C3H5+
103.11 u (C6H14O)H+

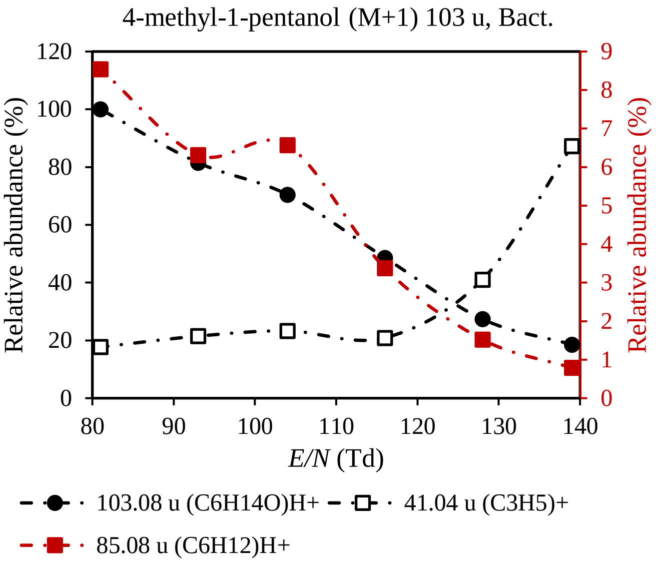
4-methyl-1-pentanol (M+1) 103 u, Bact.
Relative abundance (%)
E/N (Td)
103.08 u (C6H14O)H+
41.04 u (C3H5)+
85.08 u (C6H12)H+

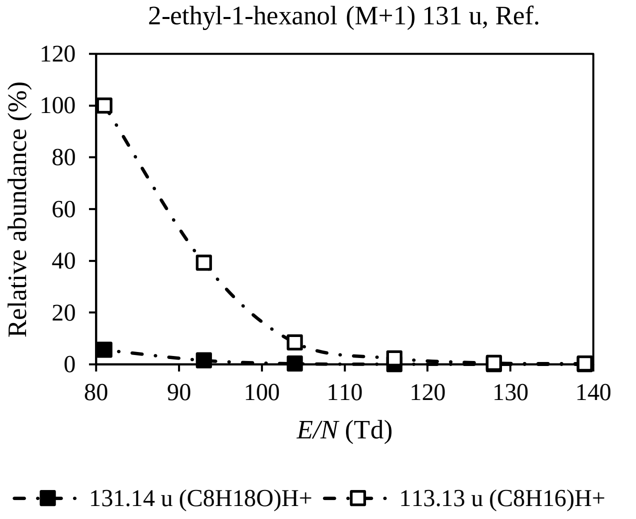
2-ethyl-1-hexanol (M+1) 131 u, Ref.
Relative abundance (%)
E/N (Td)
131.14 u (C8H18O)H+
113.13 u (C8H16)H+

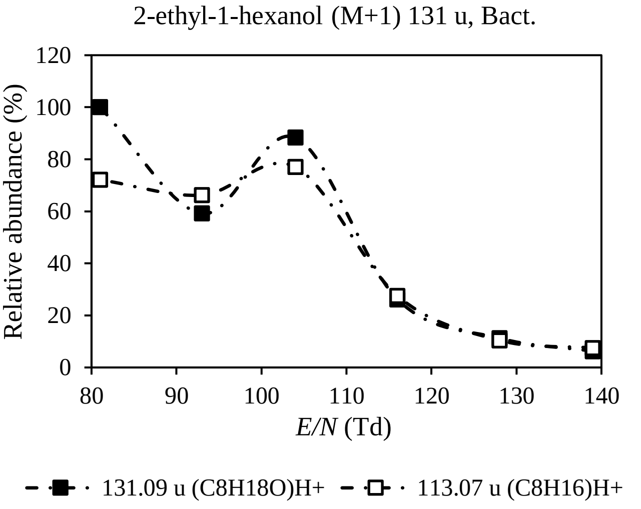
2-ethyl-1-hexanol (M+1) 131 u, Bact.
Relative abundance (%)
E/N (Td)
131.09 u (C8H18O)H+
113.07 u (C8H16)H+

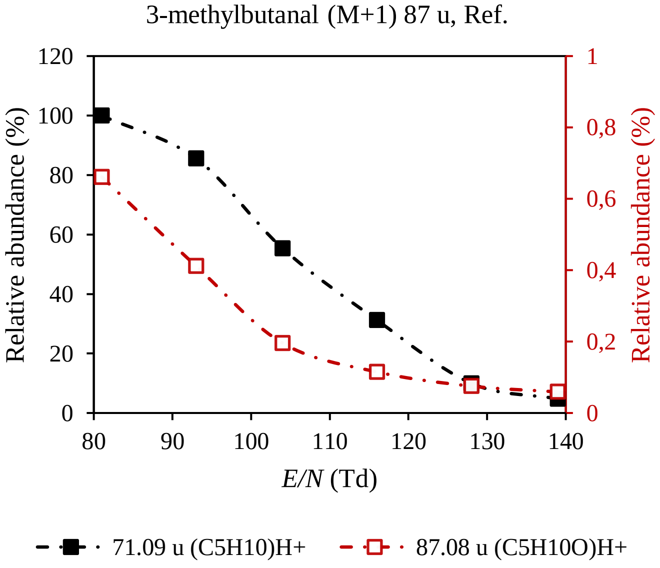
3-methylbutanal (M+1) 87 u, Ref.
Relative abundance (%)
E/N (Td)
71.09 u (C5H10)H+
87.08 u (C5H10O)H+

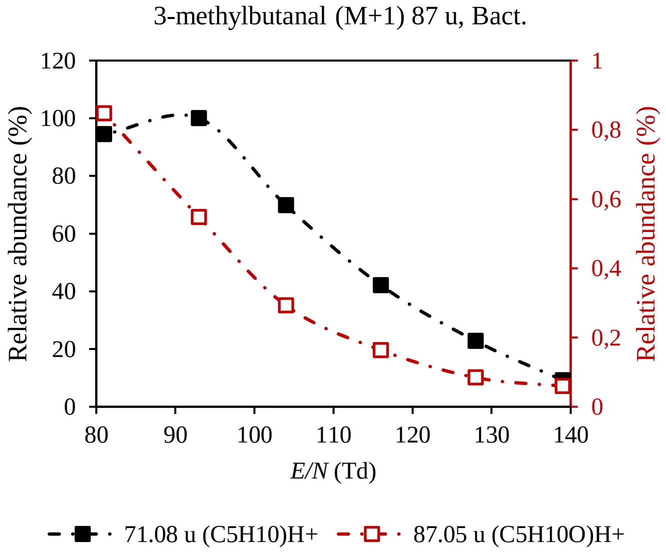
3-methylbutanal (M+1) 87 u, Bact.
Relative abundance (%)
E/N (Td)
71.08 u (C5H10)H+
87.05 u (C5H10O)H+

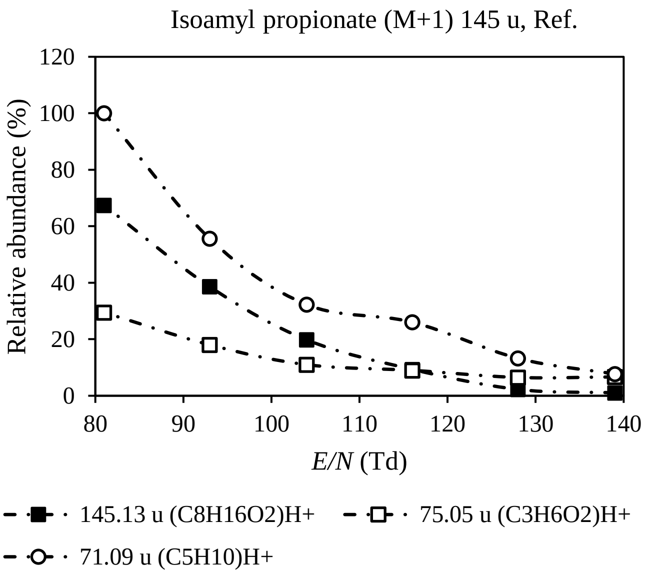
Isoamyl propionate (M+1) 145 u, Ref.
Relative abundance (%)
E/N (Td)
145.13 u (C8H16O2)H+
75.05 u (C3H6O2)H+
71.09 u (C5H10)H+

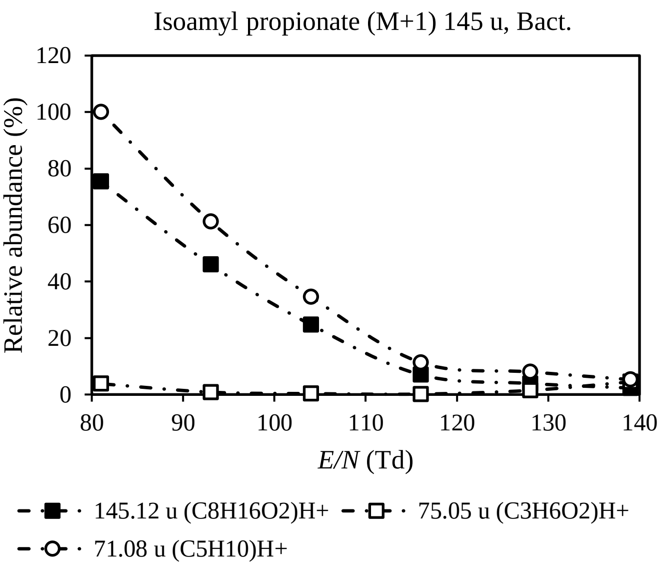
Isoamyl propionate (M+1) 145 u, Bact.
Relative abundance (%)
E/N (Td)
145.12 u (C8H16O2)H+
75.05 u (C3H6O2)H+
71.08 u (C5H10)H+

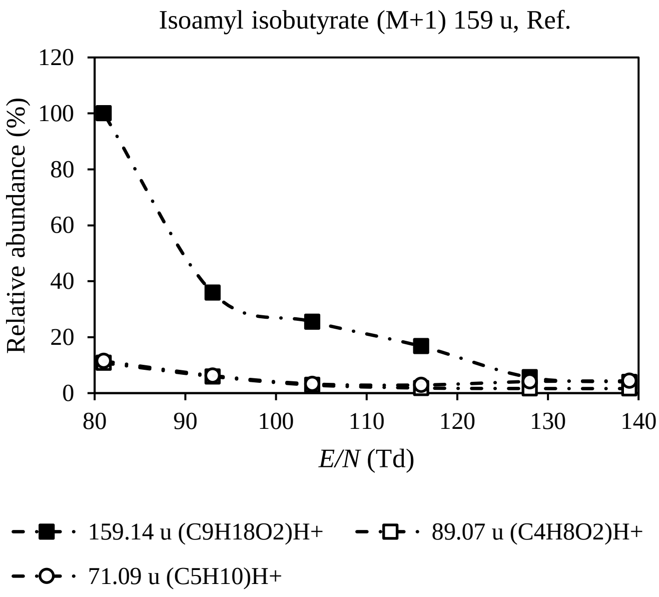
Isoamyl isobutyrate (M+1) 159 u, Ref.
Relative abundance (%)
E/N (Td)
159.14 u (C9H18O2)H+
89.07 u (C4H8O2)H+
71.09 u (C5H10)H+

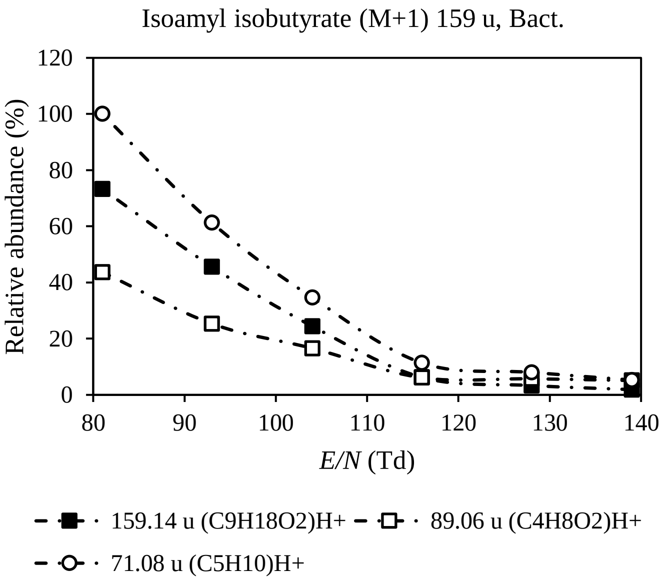
Isoamyl isobutyrate (M+1) 159 u, Bact.
Relative abundance (%)
E/N (Td)
159.14 u (C9H18O2)H+
89.06 u (C4H8O2)H+
71.08 u (C5H10)H+

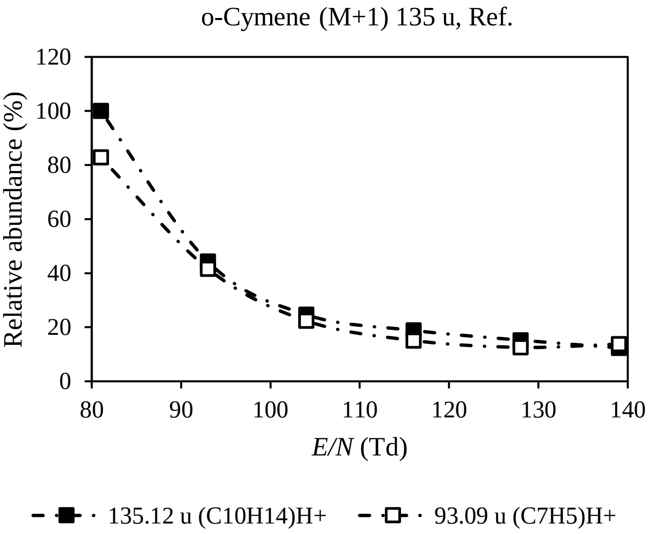
o-Cymene (M+1) 135 u, Ref.
Relative abundance (%)
E/N (Td)
135.12 u (C10H14)H+
93.09 u (C7H5)H+

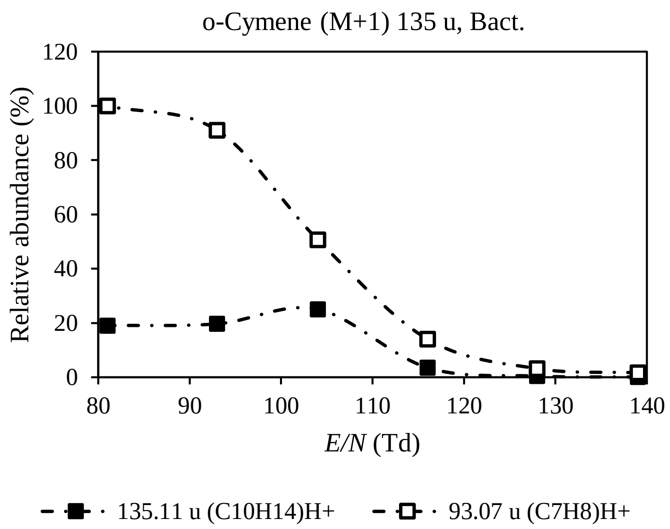
o-Cymene (M+1) 135 u, Bact.
Relative abundance (%)
E/N (Td)
135.11 u (C10H14)H+
93.07 u (C7H8)H+

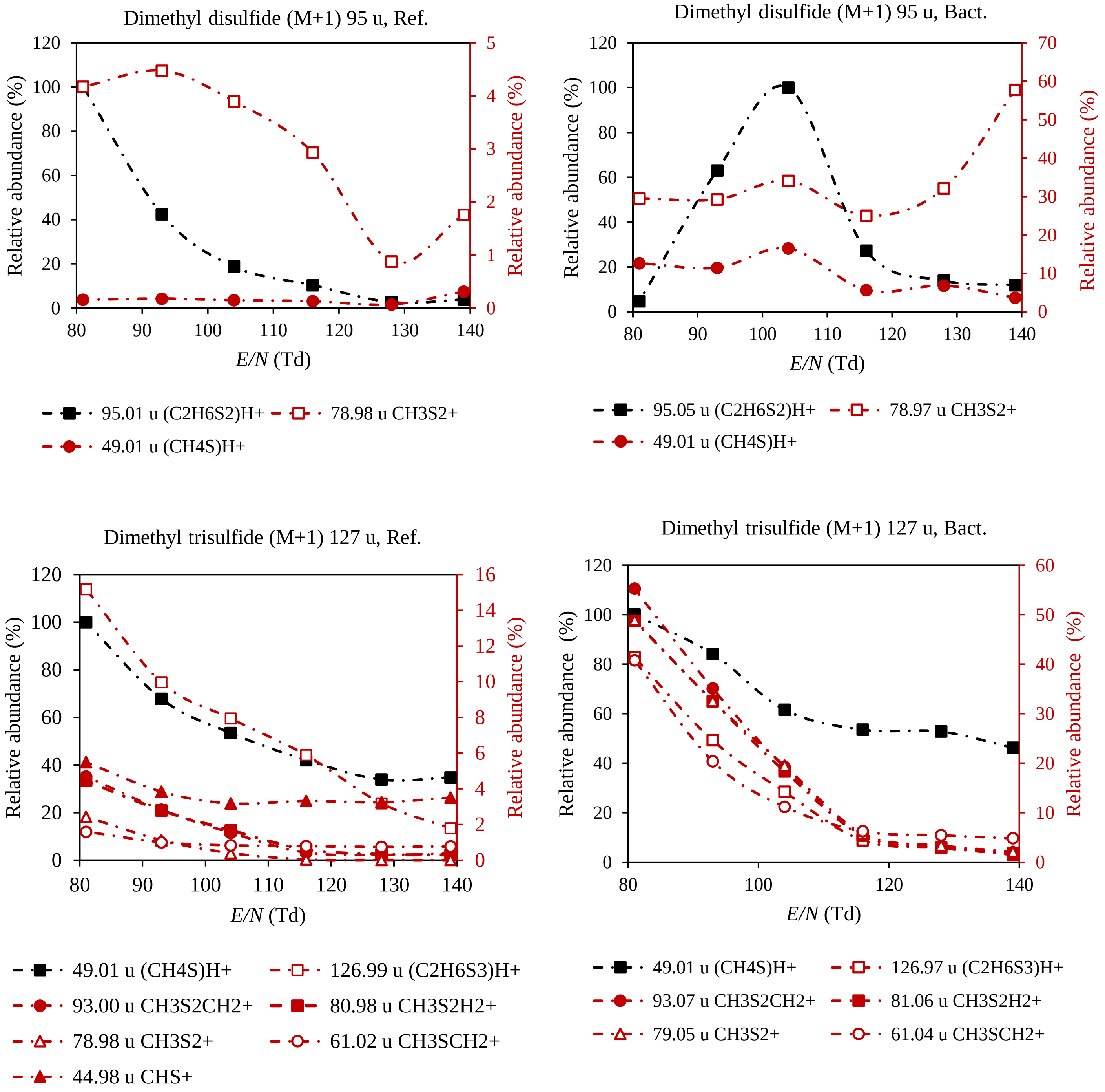

Supplementary Fig.1. Relative abundances of PTR-MS signals of the protonated molecular ion and the most important fragments. Signals are normalized to the most abundant signal (100%) in any of the different *E/N* conditions. Some diagrams have the primary y-axis indicated by black and the secondary y-axis indicated by red color.

Parent compound	Elemental composition	Calculated exact mass (u)	Accurate mass Ref. (u)	Accurate mass Bact. (u)	Difference Ref. (u)	Difference Bact. (u)
4-methyl-1-pentanol	$(C_6H_{14}O)H^+$	103,112	103,113	103,075	0,001	0,037
	$(C_6H_{12})H^+$	85,102	85,102	85,079	0,000	0,023
	$C_3H_5^+$	41,039	41,034	41,043	0,005	0,004
2-ethyl-1-hexanol	$(C_8H_{18}O)H^+$	131,144	131,142	131,092	0,002	0,052
	$(C_8H_{16})H^+$	113,133	113,134	113,066	0,001	0,067
3-methylbutanal	$(C_5H_{10}O)H^+$	87,081	87,082	87,054	0,001	0,027
	$(C_5H_{10})H^+$	71,086	71,088	71,082	0,002	0,004
Isoamyl propionate	$(C_8H_{16}O_2)H^+$	145,123	145,131	145,122	0,008	0,001
	$(C_3H_6O_2)H^+$	75,045	75,048	75,048	0,003	0,003
	$(C_5H_{10})H^+$	71,086	71,088	71,082	0,002	0,004
Isoamyl isobutyrate	$(C_9H_{18}O_2)H^+$	159,139	159,138	159,135	0,001	0,004
	$(C_4H_8O_2)H^+$	89,060	89,065	89,055	0,005	0,005
	$(C_5H_{10})H^+$	71,086	71,088	71,082	0,002	0,004
p-cymene	$(C_{10}H_{14})H^+$	135,117	135,116	135,108	0,001	0,009
	$(C_7H_5)H^+$	93,070	93,092	93,073	0,022	0,003
DMDS	$(C_2H_6S_2)H^+$	94,999	95,009	95,052	0,010	0,053
	$CH_3S_2^+$	78,968	78,982	78,968	0,014	0,000
	$(CH_4S)H^+$	49,011	49,012	49,009	0,001	0,002
DMTS	$(C_2H_6S_3)H^+$	126,971	126,986	126,972	0,015	0,001
	$CH_3S_2CH_2^+$	92,983	92,988	93,066	0,005	0,083
	$CH_3S_2H_2^+$	80,983	80,984	81,056	0,001	0,073
	$CH_3S_2^+$	78,968	78,982	78,968	0,014	0,000
	$CH_3SCH_2^+$	61,011	61,016	61,039	0,005	0,028
	$(CH_4S)H^+$	49,011	49,012	49,009	0,001	0,002
	CHS^+	44,980	44,984	45,0052	0,004	0,025

Supplementary Table 1. Elemental compositions, calculated exact masses, measured accurate masses and the mass difference are presented for each parent compound and fragment. Mass differences above the value of 0.010 u are flagged.

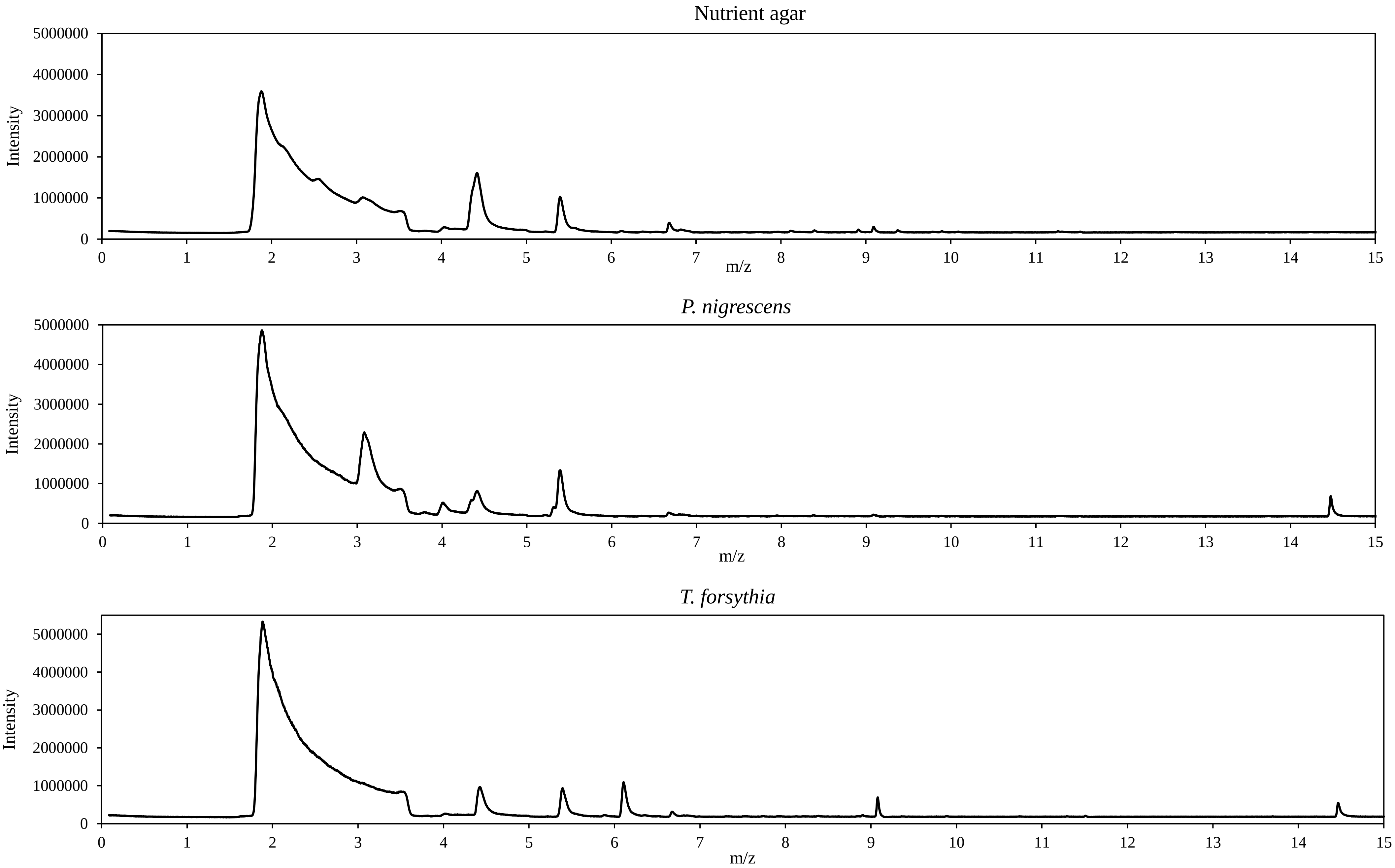
Nutrient agar
Intensity
5000000
4000000
3000000
2000000
1000000
0
0 1 2 3 4 5 6 7 8 9 10 11 12 13 14 15
m/z
P. nigrescens
Intensity
5000000
4000000
3000000
2000000
1000000
0
0 1 2 3 4 5 6 7 8 9 10 11 12 13 14 15
m/z
T. forsythia
Intensity
5000000
4000000
3000000
2000000
1000000
0
0 1 2 3 4 5 6 7 8 9 10 11 12 13 14 15
m/z

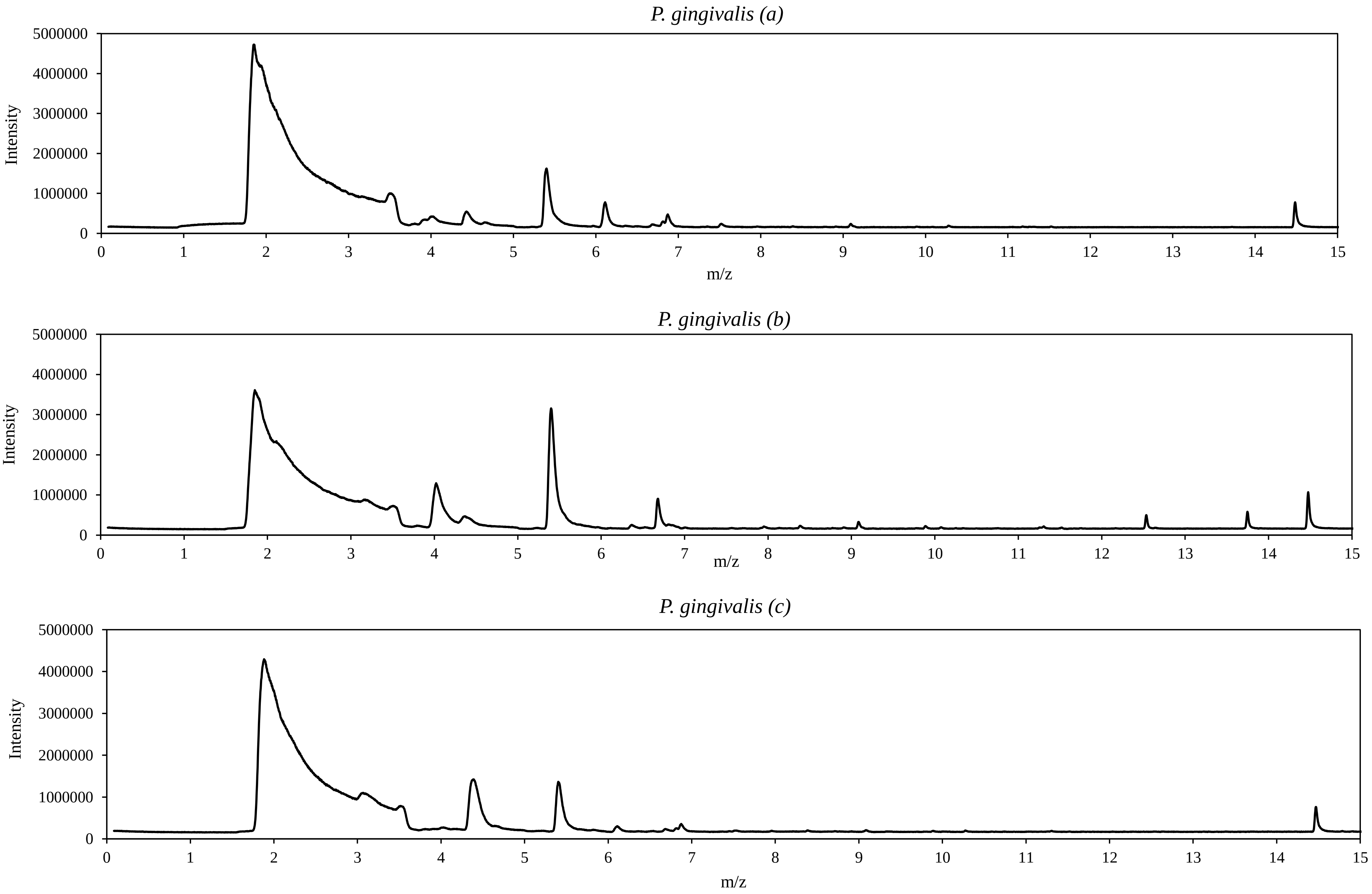

P. gingivalis (a)
Intensity
m/z
P. gingivalis (b)
Intensity
m/z
P. gingivalis (c)
Intensity
m/z

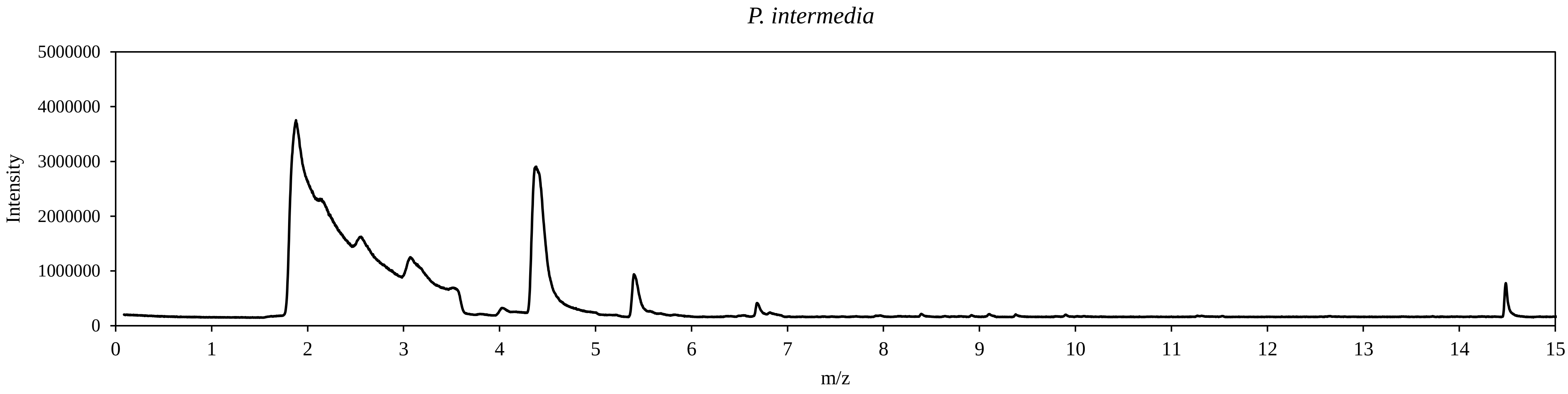

Supplementary Fig.2. Examples of chromatograms gained from bacterial and nutrient agar headspace measurements with GC-MS.

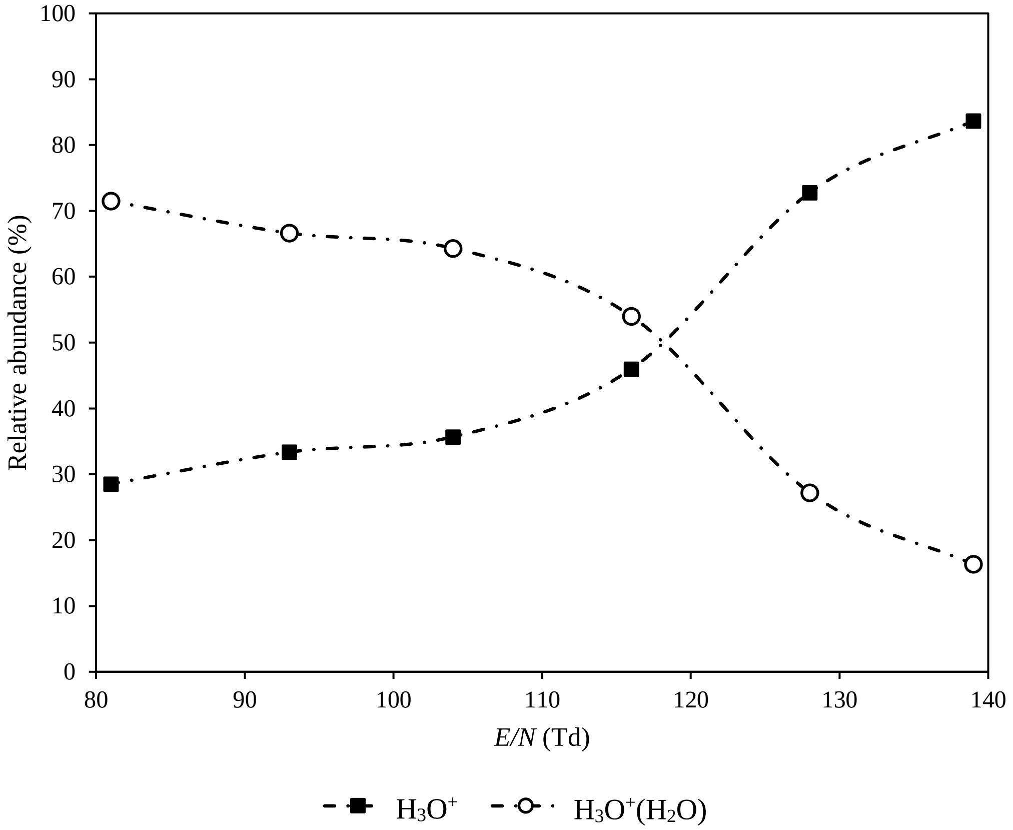

Supplementary Fig.3. Distributions of H_3O^+ ion and $H_3O^+(H_2O)$ cluster ion in the drift tube as a function of the parameter *E/N* in high humidity conditions. Relative abundances are calculated as a fraction of the total amount of ions.
